# Sleep and Wellbeing, Now and in the Future

**DOI:** 10.3390/ijerph17082883

**Published:** 2020-04-22

**Authors:** Chin Moi Chow

**Affiliations:** 1 Sleep Research Group, Charles Perkins Centre, University of Sydney, Sydney 2006, Australia; chin-moi.chow@sydney.edu.au; 2 Sydney School of Health Sciences, Faculty of Medicine and Health, University of Sydney, Sydney 2006, Australia

The processes of sleeping, eating and moving, in concert with cognition and learning, support health and life. Sleeping occurs in the rest phase, whereas the other behaviours occur in the active phase. It is largely axiomatically understood that “exercise is medicine” [1]. However, research confirms that the same dictum applies to sleep and nutrition. Indeed, sleep is medicine, as exercise and nutrition are medicine.

Wellbeing includes three aspects: life satisfaction; feelings of happiness, sadness, anger, stress, and pain; and a sense of purpose and meaning in life [2]. As such, moment to moment wellbeing, psychological or physical, is affected by activities evolving around us and can, over time, greatly impact on our health.

How does sleep directly affect wellbeing? Poor sleep and/or sleep loss (voluntarily curtailed or circumstantial) has a considerable negative impact on health and wellbeing. Zhao et al. [3] reported lower happiness in those who had short sleep duration and insomnia. We now know that short sleep increases the risk of weight gain from increased food intake in response to ghrelin *(the hunger hormone)* and decreases in leptin (*the satiety hormone*) [4]. These physiological data show that short sleep duration is related to increases in BMI and support the hypothesis that short sleep duration predicts a higher BMI. We are also aware that sedentary habits, with reduced energy expenditure and excess food intake, may also be elements that underscore this relationship between short sleep duration and obesity [5]. Garfield (2019) [6] draws attention to the fact that this relationship could be bidirectional and that obesity (using BMI cut-off points) may predict short sleep duration. Longitudinal studies using objective measures of sleep will help to clarify any causal relationship between sleep duration and BMI. 

Chronic sleep loss increases the risk for the development of diabetes, cardiovascular conditions and other pathologies [4]. Indeed, short, low-quality and mistimed sleep may threaten metabolic public health [7]. A chronic lack of sleep directly impacts on a person’s capacity to manage daily tasks, which, over time, manifests in an overwhelming sense of physical fatigue, malaise, impaired cognition and diminished wellbeing [8,9].

Sleep can deliver recovery following prolonged waking hours/sleep loss [10,11] or exhaustive exercise through many of the functions it serves. Sleep has an anabolic function. Growth Hormone (GH, an anabolic hormone) peaks [12], whereas cortisol (a catabolic hormone with capacity for muscle catabolism and bone demineralization) concomitantly dips to its nadir [13], within the first two sleep cycles. Thus, the temporal associations between GH/cortisol and sleep furnish a pathway for tissue restoration and restorative sleep, contributing to overall wellbeing. 

The importance of recovery is illustrated clearly in competing athletes, who have an increased sleep need to meet the energetic, metabolic and vascular demands of exercise training. Insufficient sleep may be a result of poor sleep due to psychological or social stresses [14], apart from physical demands. Many athletes have reported difficulty in initiating sleep, night-time waking and difficulty in getting up in the morning [15,16]. Thus, the strategic planning of recovery sleep includes napping, sleep extension for sleep deprived athletes and promoting “best possible” sleep hygiene to facilitate regular sleep-wake patterns [17], which are cardinal elements for optimising sports performance. 

Sleep recovery is not only physically important for athletes but for the general population. During slow wave sleep, toxic metabolic wastes (e.g., beta-amyloid proteins, a biomarker of Alzheimer disease) are cleared [18], GH is released [12] and glycogen granules are accumulated in the brain [19,20], serving as a fuel reserve for the brain at times of neuronal activation. 

Sleep can also affect psychological wellbeing. Sleep serves a synthetic function of new nerve structures (e.g., of synapses) that follows learning and memory consolidation [21]. This memory feature is particularly critical for our existence, i.e., the ability to recognise, learn, acquire motor procedures, map physical space, develop language expression and permit creativity. Memories give life purpose and meaning.

Sleeping difficulties are common. Difficulties in initiating or maintaining sleep or waking too early may arise from a variety of factors including sleeping too cold/hot, medication, alcohol and caffeine use, and pain. Eating within 3 h of bedtime is positively associated with nocturnal awakening [22]. Furthermore, different psychiatric disorder categories are associated with different sleep complaints in pre-schoolers [23]. 

Some sleep issues may be mitigated by choosing an appropriate pillow type, e.g., with an appropriate pillow neck and side height [24], and sleepwear fabric type, e.g., sleeping in wool promoted improved sleep [25,26]. The food type consumed may also help to promote sleep onset, e.g., a high glycemic index meal shortened sleep onset compared to a low glycemic index meal [27]. 

The question remains “why can’t some people sleep”? Pressing issues, especially when acute and psychological in nature, can often deter sleep. *The city doesn’t sleep* [28] highlighted social factors such as socio-economic status, safety/security and future insecurity as weighty social divides of sleep disparity, suggesting they were sources of stress and anxiety arising from daily living. Hang-ups (not being able to let go) at bedtime postpone sleep. Stress, worries and anxiety culminate in heightened arousals with consequential increases in the pulsatility of nocturnal cortisol, when only few, regular cortisol pulses should exist [29]. 

Together, stress and anxiety are significant sources of sleeping difficulties. Recent developments in endocannabinoid signalling, which has a key role in the regulation of stress responses and emotional learning [30], may signpost and prioritise research opportunities in this field. The signalling molecule 2-arachidonoylglycerol (2-AG) appears to protect against stress [31]. In the presence of acute stress exposure, the failure of 2-AG signalling would result in the strengthening of anxiety-producing connections between the amygdala and prefrontal corticies [31]. This mouse model research is very encouraging, since the identification of anxiety-producing signalling pathways may provide an insight into future treatment possibilities.

Different strategies are available for managing sleep loss in shift work. Sleep medicine has come a long way due to increased public awareness, as highlighted by the study of Savic et al. (2019), who reported that the nurses in their study had already adopted health-promoting coping strategies with their night shifts [32], including health practices of physical activity, healthy eating, engaging with social support and leisure, mindfulness, managing time, and work-related coping strategies. Nevertheless, the online platforms of mHealth [33] and cognitive behavioural therapy for insomnia (CBTi) [34] are resourceful, easily accessible materials for shift workers. Forest therapy (originating in the 1980s in Japan for stress reduction) that improved sleep efficiency in cancer patients [35] may be explored in shift workers in future studies. 

Finally, sleep is as individual as the individual. Sleeping behaviour is an individual practice. An individualised approach to treating sleep difficulties is a step towards personalised medicine for optimising health and both physical and psychological wellbeing. 
